# Integrating BWM and modified VIKOR for age-friendly community park assessment in the context of mental health

**DOI:** 10.3389/fmed.2025.1715370

**Published:** 2025-11-19

**Authors:** Zhi-Hao Zhang, Yi-Fan Zhang, Cheng-Cheong Lei

**Affiliations:** 1 School of Knowledge Science, Japan Advanced Institute of Science and Technology, Ishikawa, Japan; 2 College of Design and Innovation, Tongji University, Shanghai, China; 3 Faculty of Arts and Design, Macao Polytechnic University, Macau, Macao SAR, China

**Keywords:** healthy aging, community parks, mental health, best-worst method, VIKOR

## Abstract

**Background:**

Late-life depression is a growing public health challenge in aging societies. Community parks are increasingly recognized as protective environments, yet evidence on which specific attributes most effectively alleviate depression among older adults remains limited.

**Methods:**

This study proposed a hybrid framework combining the Best-Worst Method (BWM) and a Modified VIKOR approach. Eight criteria (Access points, Pathways, Facilities for different activities, Open space, Amenities, Natural features, Sunlight, Incivilities) were identified through literature review. Twenty-three experts conducted BWM pairwise comparisons, with 13 valid responses retained after consistency checks. Nine community parks in Zhuhai, China, were then evaluated using gap scores and ranked via Modified VIKOR.

**Results:**

The evaluation revealed that open space (16.26%), facilities for different activities (14.27%), and public amenities (13.50%) were the most influential in reducing depression risk. Rankings showed notable disparities among the nine parks. Improvement priorities focused on expanding spaces for exercise and social interaction, upgrading seating, lighting, and waste facilities, and gradually enhancing vegetation and environmental order. Access and pathway conditions were generally adequate, requiring only localized adjustments.

**Conclusion:**

Community park environments contribute to late-life depression alleviation through differentiated mechanisms. The BWM-Modified VIKOR framework provides an evidence-based tool for prioritizing age-friendly park renovations under limited resources. Priority should be given to upgrading activity facilities, open spaces, and amenities.

## 1 Introduction

The global population is undergoing rapid aging (1). According to United Nations projections, by 2050, individuals aged 60 and above will account for 21.5% of the global population, reaching 2.1 billion (2). As aging accelerates, the associated health burden is also intensifying (3, 4). Among these, depression is one of the most prevalent mental disorders in older adults, often triggered by limitations in physical, emotional, cognitive, and sensory functions that lead to disability (5, 6). In addition, physiological decline, role transitions such as retirement and widowhood, and insufficient self-care among those living alone substantially increase the risk of depression (7, 8). However, late-life depression is often misinterpreted as normal aging or an acceptable stress response, delaying recognition and intervention (9). Consequently, the prevalence of depression and suicide among older adults remains high and continues to rise (8, 10). These outcomes are symptomatic of multiple comorbid health problems, typically manifested as anhedonia, insomnia, appetite loss, and even cognitive changes (9, 11). Such conditions not only severely impair individual wellbeing but also impose a sustained burden on healthcare systems (10). In China, late-life depression is particularly shaped by cultural and familial structures. Concerns about becoming a burden to the family often exacerbate anxiety, while large urban–rural disparities result in limited awareness and expression of mental disorders (12).

This phenomenon is not confined to the individual level but is closely tied to the social environment and structural factors (13). These exert profound influences on the mental health of older adults, especially under the pressures of rapid urbanization (14). Restricted mobility due to physical conditions, low adaptability to the environment, and inadequate external social support often delay responses to high-risk contexts (8, 15). Hence, urban environments play a vital role in mitigating depression among older populations. Within this context, landscape ecology emphasizes the regulatory effects of external environmental features, particularly the physical environment (8).

Empirical evidence has demonstrated that exposure to greenery and natural environments offers varying degrees of psychological benefits for older adults (16–19). These effects are primarily realized through experiences at both psychological and behavioral levels. In environmental psychology, the Stress Recovery Theory (SRT) and Attention Restoration Theory (ART) have been widely employed to explain such restorative mechanisms (20, 21). SRT posits that elderly individuals experience emotional relief and mental relaxation in tranquil park spaces, where the safe and gentle atmosphere of nature helps alleviate stress and mitigate negative emotions such as depression (22, 23). ART, on the other hand, suggests that park environments provide opportunities for individuals to withdraw from daily hassles and engage in softly fascinating experiences, allowing older adults to restore their attentional capacities depleted by prolonged tension, thereby regaining calmness and positive affect through effortless perception and exploration (19).

Beyond psychological restoration, natural environments also exert compound effects on social interaction and physical activity. Specifically, parks provide venues for gatherings and social contact among older adults, strengthening social support networks and effectively reducing feelings of loneliness and depression (24, 25). Meanwhile, natural settings promote physical activity, which produces indirect benefits—green exercise, walking, and leisure participation not only enhance physical functioning and engagement but also reinforce cognitive performance and immune responses (20, 21). Together, these mechanisms form an integrated system in which emotional restoration, social bonding, and activity motivation interact synergistically. Among the diverse urban green space typologies, neighborhood-level green areas that are easily accessible and integrated into daily life have been increasingly recognized for their potential to support mental wellbeing in aging populations (26).

With technological progress and changes in residential forms, green spaces in some Asian cities have evolved into diverse configurations comprising public, semi-public, and private domains (27, 28). Within this context, community parks—typically small or medium-sized green spaces located within residential neighborhoods and primarily serving nearby residents—represent an important typology in the Asian urban park system (29). Owing to their proximity and environmental quality, these parks serve as the main medium for daily contact with nature (30). Their high accessibility transforms human–nature interaction from an occasional occurrence into a habitual experience, subtly buffering life stress and emotional burdens (8, 31). Furthermore, their open and shared spatial attributes provide individuals with opportunities for social interaction and companionship, thereby preventing the accumulation of isolation (32). The functional design and low entry threshold of such parks further ensure active participation of elderly users, enhancing their sense of self-efficacy and overall quality of life (33). Through this intertwined relationship among natural environment, social connection, and behavioral practice, community parks not only compensate for the absence of private green spaces but also demonstrate a unique and irreplaceable value in alleviating and improving late-life depression.

Despite their potential value, research focusing on the role of community parks in supporting older adults' mental health remains limited (34–38). Most existing studies emphasize overall urban greenness or general perceptions of residents, while empirical evidence concerning community parks in high-density urban settings and their relationship to geriatric depression is still scarce (39–42). Current research often relies on single indicators such as green coverage, accessibility, or visual greenery, lacking a comprehensive understanding of the parks' physical environmental characteristics. In particular, the relative influence and improvement priorities of different spatial features, such as open space configuration, facility maintenance, path connectivity, and sensory design, within specific cultural contexts have not been systematically explored.

Moreover, many existing assessments employ simple additive models based on fixed-weight audit tools, such as the Neighborhood Green Space Tool (NGST) (43). Although such instruments can provide an overall evaluation of environmental quality, their predetermined weights often fail to reflect socio-cultural or spatial contextual variations, thereby reducing the practical and policy relevance of the results (43–45). In recent years, Multi-Criteria Decision-Making (MCDM) approaches based on expert judgment have attracted increasing attention in urban environmental evaluation (42, 43, 46). By incorporating multidisciplinary expert knowledge into the weighting process, MCDM methods can better account for contextual diversity, resulting in more targeted and applicable outcomes. However, applications of such methods to the intersection of community park evaluation and late-life depression remain scarce.

To address these gaps, this study establishes an integrated evaluation framework centered on the BWM and Modified VIKOR approach. Environmental criteria of community parks were derived through literature review, followed by weight determination using BWM and comprehensive ranking and diagnosis of nine community parks in Zhuhai City using Modified VIKOR. The BWM is characterized by structural simplicity, low data requirements, and computational efficiency, while the Modified VIKOR method, grounded in the principle of compromise programming, balances overall performance with individual weaknesses through reference to an ideal solution—revealing both global advantages and priority areas for improvement (47, 48).

In summary, this research develops a BWM–Modified VIKOR–based framework that bridges the theoretical and methodological gap between community park environmental features and elderly depression studies. The proposed model not only expands the analytical perspective at the intersection of geriatric health and urban design but also provides a practically oriented evaluation tool and decision reference for optimizing age-friendly community parks.

## 2 Literature review

### 2.1 Evaluation of physical environmental quality in parks

In the evaluation of physical environmental quality in parks, a range of objective audit tools have been developed, such as the Community Park Audit Tool (CPAT), Public Open Space Desktop Auditing Tool (POSDAT), Environmental Assessment of Public Recreation Spaces (EAPRS), and PARK Tool (49–53). These instruments, however, were largely designed based on the spatial morphology of low-density Western cities and typically rely on fixed-weight scoring systems to quantify the physical environment (49, 54–56). In the context of high-density and culturally diverse Asian cities, such tools fail to adequately capture the complex spatial configurations and diversified user needs that characterize local urban settings. Moreover, the use of simple additive weighting to generate composite scores often results in descriptive assessments rather than decision-oriented analyses, thereby limiting their capacity to inform strategic planning and spatial optimization for park improvement.

To overcome these limitations, recent studies have introduced MCDM frameworks to enhance both the adaptability and decision-oriented nature of evaluation models. These approaches incorporate expert judgment, enabling a more context-sensitive assessment of complex environments and cultural variations. For example, the AHP–TOPSIS framework ranks park alternatives based on normalized data and has been applied to examine the relationship between urban green space quality and visitors' psychological wellbeing (57). Meanwhile, the DEMATEL–DANP–VIKOR system integrates inter-attribute relationships to uncover the formation mechanisms of perceived restorative capacity, identifying critical pathways that influence recovery experience and emphasizing a systemic and compromise-oriented perspective (58). Srdjevic et al. (36) further compared the applicability and stability of AHP and BWM in park quality evaluation, concluding that BWM demonstrates higher consistency and computational efficiency in weight assignment (36). Other studies have employed the DANP–Modified VIKOR model to develop continuous improvement strategies for public open spaces, identifying key influencing factors and proposing optimization pathways for age-friendly park environments (59). Similarly, Li et al. integrated rough set theory to analyze combinational scenarios of urban green landscape features, identifying landscape element configurations that positively contribute to the perceived recovery of individuals with depressive symptoms (60).

Taken together, these approaches highlight complementary advantages: BWM excels in reducing cognitive burden and enhancing robustness during the weighting process (61), whereas Modified VIKOR offers dynamic compromise and multi-objective balancing capabilities, revealing the potential for improvement across psychological health dimensions (59). However, research integrating both methods remains scarce—particularly in the context of evaluating elderly-oriented park environments from a mental health perspective—indicating an urgent need for further exploration in this domain.

### 2.2 Construction of evaluation framework

To ensure the reliability and contextual relevance of the proposed indicator system, this study followed a three-step process:

(a) **Selection of a baseline audit framework**

The NGST was chosen as the basis for community park audits for the following reasons. First, audit tools that do not specifically incorporate older adults, such as QUINPY (56) and PARK (49), were excluded. Second, in this study, community parks are defined as small-scale open green spaces surrounding residential neighborhoods and serving local residents (also referred to as “neighborhood green spaces”). These parks differ from large-scale urban parks and other public spaces in terms of both experiential attributes and facility configurations (62, 63). Consequently, design tools that are unsuitable for small-scale community parks, such as RECITAL (55) and NEST (54), were excluded.

(b) **Empirical validation and theoretical justification**

Evidence from multiple studies across Europe, Asia, and North America consistently demonstrates that urban parks play a vital role in relieving depressive symptoms, fostering positive emotions, and improving mental health (64–66). Specifically, several spatial attributes have been repeatedly linked to psychological outcomes:

*Access Points*: Well-distributed park entrances not only shorten walking distances and enhance accessibility but also promote more frequent and spontaneous park visits, which are crucial for sustaining psychological wellbeing among older adults. Improved accessibility reduces perceived physical barriers, thereby lowering psychological stress and the risk of mood disorders (67, 68).

*Pathways*: Park pathways serve as both functional and psychological corridors that facilitate walking, slow movement, and meditative activities known to regulate emotions and alleviate depressive symptoms. Wide, flat, and well-equipped walkways provide a sense of safety and continuity, encouraging longer stays and repetitive use, which reinforce the restorative benefits of nature exposure. Beyond mobility, the design quality of paths—such as surface texture, shading, and visual enclosure—plays a pivotal role in shaping emotional regulation and perceived tranquility (69, 70).

*Open Spaces*: Studies indicate that larger park areas are generally associated with lower depression risk, although this benefit weakens when non-vegetated areas (e.g., sports courts) occupy a high proportion (64). Moderate spatial complexity and higher green patch density foster positive emotions (8), whereas excessive visual or structural complexity may reduce dwell time and weaken restorative effects (71). Parks with clear landscape layering and balanced vegetation stimulate relaxation and calmness (72), while excessive clearing in pursuit of tidiness or safety can diminish naturalness and biodiversity, thereby undermining psychological restoration (73).

*Facilities for Different Activities*: The quantity and quality of exercise equipment are directly related to older adults' sense of safety and improvement in depressive symptoms. Well-maintained facilities and clearly defined park edges not only reduce perceived risks but also help slow the progression of depressive tendencies (71, 74).

*Amenities and Natural features*: Well-maintained benches, pavilions, toilets, lighting, and signage alleviate monotony and increase perceived comfort, while rich vegetation, water features, and flowering plants help individuals temporarily detach from negative emotions, prolong stays, and enhance restoration (71, 74).

*Incivilities*: Accessibility, non-slip surfaces, proper waste management, clear boundaries, and good maintenance improve perceived safety and reduce depressive tendencies, whereas derelict buildings, noise, and vandalism may exacerbate psychological distress (71, 75–79).

(c) **Refinement and supplementation of criteria**

Building upon NGST, the criteria system was revised and expanded based on recent empirical findings. Following the work of Li et al. (60) two additional criteria were incorporated: *public art or installation* (under the *Amenities* criterion) and *sunlight*.

*Public art or installation* contributes to depression alleviation through both physiological mechanisms (e.g., endorphin release and aesthetic pleasure) and social mechanisms (e.g., promoting social interaction and communication skills) (80). Adequate *Sunlight* exposure influences behavior patterns in nuanced ways: strong daylight encourages outdoor observation and engagement with nature, while softer lighting environments foster intimate conversation—both contributing to emotional stability and reduced depression risk (71, 81).

At the same time, criteria such as *Pedestrian Crossings* or *Shortcuts* were not included in the core framework due to the current lack of direct empirical evidence linking them to mental health outcomes among older adults. Nonetheless, prior studies at the macro level have confirmed that such elements are indeed associated with walking accessibility, safety, and psychological wellbeing (82), and should therefore be considered in future extensions of the model.

Based on this literature review, the present study constructed a measurement scale to assess the effects of community park environments on depression alleviation among older adults, as shown in Table 1.

**Table 1:** Criteria for evaluating community parks.

**Criteria**	**Description**	**References**
Access points	Number of access points; shortcuts connecting areas; pathway availability and quality	(67, 68, 90)
Pathways	Number and quality of pathways	(70)
Facilities for different activities	Number and quality of equipment for physical and social activities	(71, 74)
Open space	Quality and provision of open space	(8, 66, 71)
Amenities	Seating, litter bins, dog bins, lighting, public art or installations	(33, 80, 87, 88)
Natural features	Quantity and quality of vegetation (trees, flowers, plants) and water features	(71, 87, 89)
Sunlight	Moderate natural light exposure	(71, 81)
Incivilities	Signs of disorder, including litter, alcohol debris, graffiti, vandalism, dog mess	(71, 75–79)

## 3 Methods and materials

**Figure 1:**
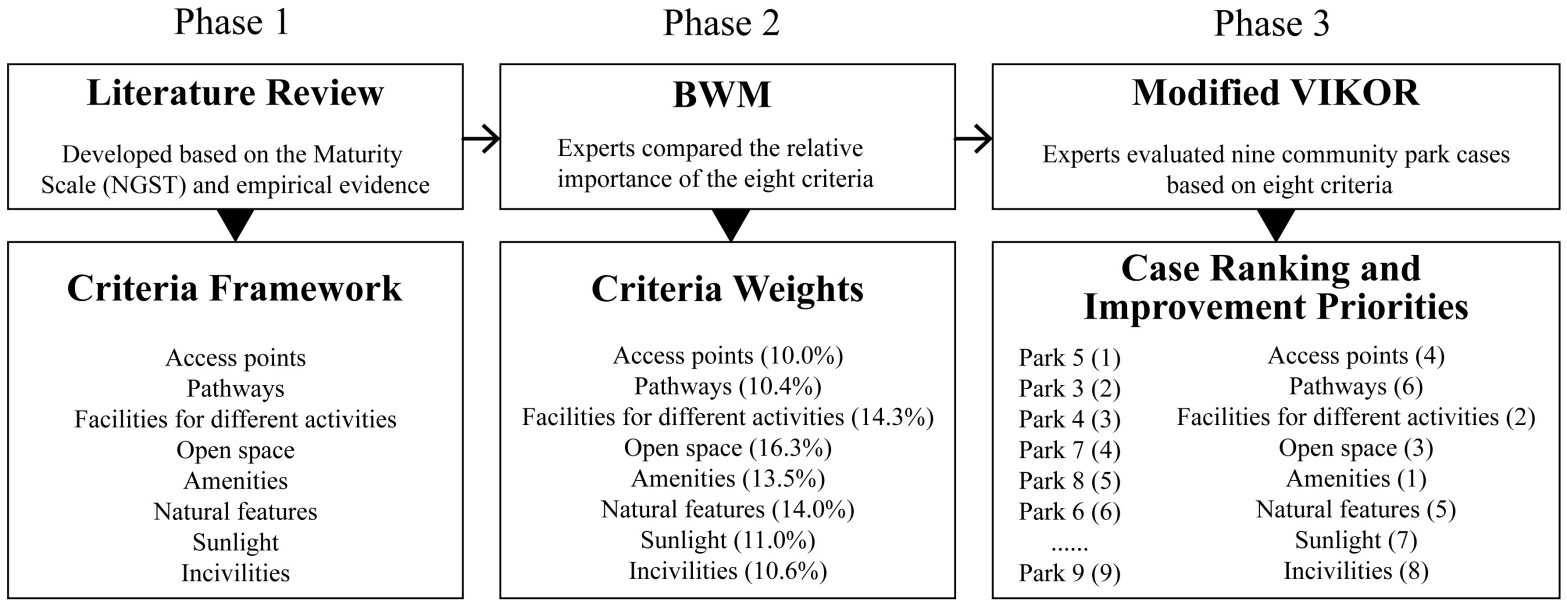
Methodology framework.

This study employed the BWM–Modified VIKOR model, a MCDM approach. The research process consisted of three main stages. In the first stage, a literature review was conducted to extract eight evaluation criteria from the physical environmental features of community parks that may influence the alleviation of depression among older adults. In the second stage, the BWM was applied to determine criteria weights. Experts compared the relative importance of the ‘best' and ‘worst' criteria to calculate the weights of all criteria. In the third stage, the Modified VIKOR method was employed to conduct a comprehensive evaluation and ranking of nine representative community park cases. Accordingly, a comprehensive evaluation framework was established to assess the potential of community parks in alleviating depression among older adults, based on expert experiential judgment. The overall methodological framework is presented in Figure 1.

### 3.1 Empirical case

The empirical study selected nine community parks in Xiangzhou District, Zhuhai, as research objects. In this district, the proportion of residents aged 60 years and above has exceeded one-tenth of the total population and continues to grow (83). At the same time, Zhuhai has implemented a specialized plan for elderly care services, promoting the concept of ‘community-based, nearby elderly care', with an emphasis on environmental optimization, greening, and age-friendly renovation. This reflects the urgent demand for high-quality public spaces (84). Against this background, nine representative cases with appropriate heterogeneity were deliberately selected, covering a broad spectrum of community parks in terms of scale and performance distribution across multiple criteria. These cases are therefore suitable for demonstrating the applicability of the proposed evaluation framework.

During data collection, domain experts were recruited to complete both the BWM questionnaire and the park criteria evaluation survey in August 2025. The inclusion criteria were as follows: (a) a relevant background in urban planning and design, public health, environmental psychology, or public art, along with adequate expertise in community park design and geriatric psychology; (b) a master's degree or above in a related field; and (c) practical or academic experience related to community parks, geriatric medicine, or similar topics, including participation in related projects or publications. In total, 23 experts were recruited, and their demographic and professional characteristics are summarized in Table 2.

**Table 2:** Profile of participating experts.

**Characteristic**	**Categories**	**Number**	**Proportion (%)**
Gender	Male	16	69.6
	Female	9	30.4
Age (years)	20–30 (inclusive)	23	100
Education	Master	13	56.5
	Doctor	10	43.5
Field of expertise	Urban planning and design	19	82.6
	Design, public health, and environmental psychology (interdisciplinary)	3	13.0
	Public art	1	4.4
Years of professional experience	1–3 (inclusive)	9	39.1
	3–6 (inclusive)	10	43.5
	≥6	4	17.4

All 23 experts completed the BWM questionnaire. A relatively strict consistency threshold of 0.1 was applied, and 13 experts were retained for the BWM weight calculation; the questionnaire structure and computational procedures are detailed in the following section. To assess the performance of the nine community park cases, an online auditing approach was employed. The research team conducted on-site photography at 50-meter intervals, capturing images from the front, back, left, and right directions at each point, and compiled all photographs into an online package for expert review. Based on criteria descriptions and professional judgment, experts provided quantitative ratings (1–10) for eight criteria and the overall park perception. This remote audit approach has been widely adopted in previous studies and is considered a reasonable and robust evaluation method (85, 86).

### 3.2 BWM

**Figure 2:**
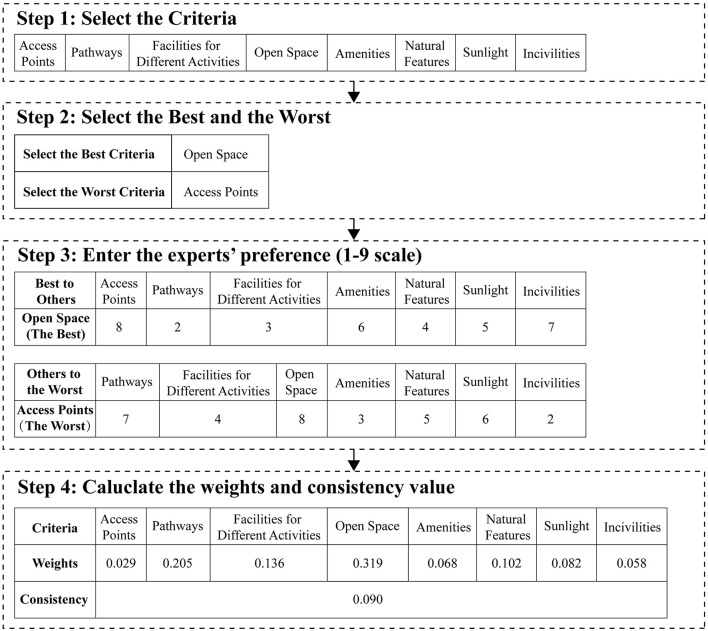
The procedure of BWM.

The detailed questionnaire design and calculation procedure of the BWM are shown below (see Figure 2):

**Step 1:** Attribute selection. Based on a literature review, the attributes to be analyzed were identified, including access points, pathways, facilities for different activities, open space, public amenities, natural features, sunlight, and incivilities.

**Step 2:** Experts determined the best and worst attributes.

**Step 3:** Each expert was asked to use a 1–9 scale to evaluate (a) the relative importance of the ‘best attribute' compared with all other attributes (Best-to-Others *a*_*Bj*_), and (b) the relative importance of each attribute compared with the ‘worst attribute' (Others-to-Worst *a*_*jW*_). Experts then provided preference scores for all attributes (including the worst attribute).

**Step 4:** The optimal weights of all attributes ($w_{1}^{*},\;w_{2}^{*}$, …, $w_{n}^{*}$) were subsequently calculated. The objective was to minimize the maximum absolute deviation across all attributes *j*. This yields the following min–max optimization model:


$$\begin{array}{l} \text{min max }\left\{\left|w_{B}-a_{Bj}w_{j}|,|w_{j}-a_{jW}w_{W}\right|\right\} \end{array}$$ (1)

$$\begin{array}{l} \text{s}.\text{t}.\underset{j}{\sum }w_{j}=1,\;w_{j}\geq 0,\text{ for all }j \end{array}$$ (2)

To improve computational efficiency and stability, the nonlinear model was transformed into a linear programming form *min ξ*^*L*^ *s*.*t* (62):


$$\begin{array}{l} |w_{B}-a_{B_{j}}w_{j}|\leq \xi^{L},forall\;j \end{array}$$ (3)

$$\begin{array}{l} |w_{j}-a_{jW}w_{W}|\leq \xi^{L},for\;all\;j \end{array}$$ (4)

$$\begin{array}{l} \underset{j}{\sum }w_{j}=1,\;w_{j}\geq 0,for\;all\;j \end{array}$$ (5)

Solving the model produces the optimal weight vector ($w_{1}^{*},\;w_{2}^{*}$, …, $w_{n}^{*}$) along with the consistency values ξ^*L*^. A consistency value ξ^*L*^ closer to 0 indicates higher internal consistency in expert evaluations (63). Among the 23 expert evaluations, 10 experts produced consistency values ξ >0.1, and their judgments were excluded for insufficient consistency. The weights of the eight attributes were calculated for each of the remaining 13 experts, and the average values were taken as the final distribution of criteria weights, as shown in Table 3.

**Table 3:** The results of BWM.

**Criteria**	**Weights from BWM (%)**
Access points	10.0
Pathways	10.4
Facilities for different activities	14.3
Open space	16.3
Amenities	13.5
Natural features	14.0
Sunlight	11.0
Incivilities	10.6

### 3.3 Modified-VIKOR

This study applied a 10-point scale to evaluate nine community parks in Xiangzhou District, Zhuhai (*A1,A*_2_*,…,A9*). The evaluation criteria included eight dimensions: access points, pathways, facilities for different activities, open space, public amenities, natural features, sunlight, and incivilities (*n* = 8). For Park *A*_*k*_ under criterion *j*, the performance score is denoted as *f*_*kj*_. The relative importance of criterion *j* in the BWM is denoted as *w*_*j*_, where *j*=1,2,…, *n*.

**Step 1:** Using a 10-point scale, decision-makers set the aspired value $f_{j}^{aspired}$ as 10 and the worst value $f_{j}^{worst}$ as 1. Raw performance scores were then transformed into normalized gap scores *r*_*kj*_, reflecting the degree of deviation of each park from the ideal state. The normalization formula is:


$$\begin{array}{l} r_{kj}=\left(\left|f_{j}^{\text{aspired}}-f_{kj}\right|\right)/\left(\left|f_{j}^{\text{aspired}}-f_{j}^{\text{worst}}\right|\right)=\left(\left|10-f_{kj}\right|\right)/\left(10-1\right) \end{array}$$ (6)

**Figure 3:**
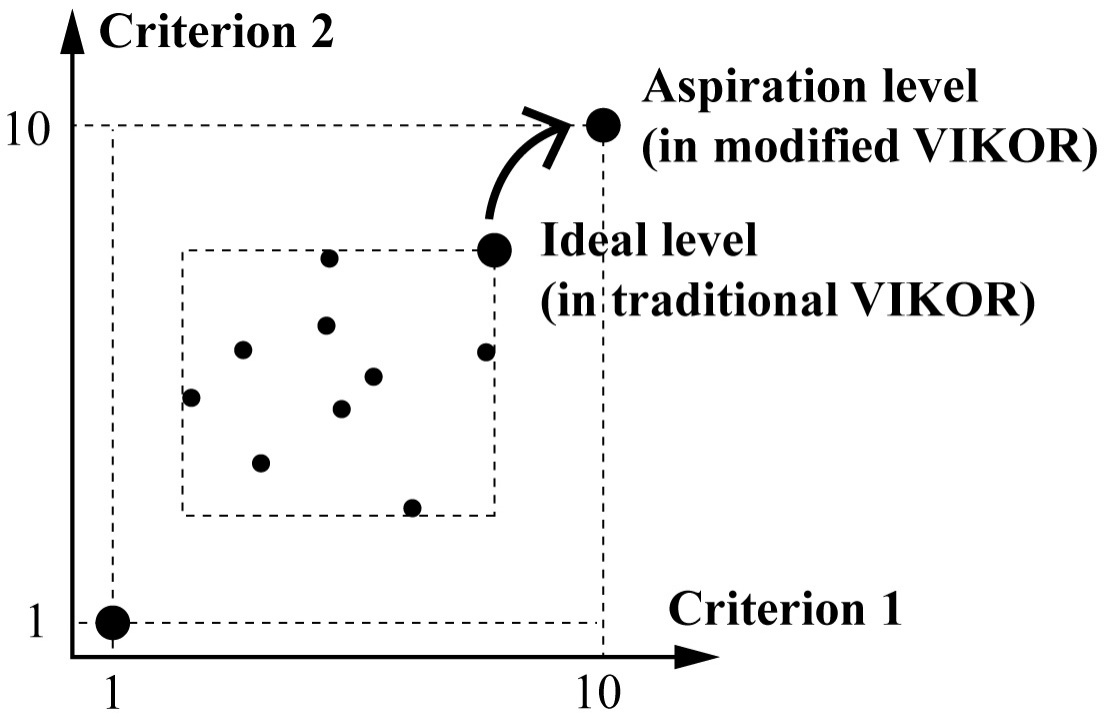
The main difference between traditional VIKOR and Modified VIKOR.

This modification, which defines the questionnaire scale directly as the decision space rather than using the performance of criteria as the decision space, enables the modified VIKOR to evaluate alternatives against a theoretical ideal instead of a relative best within the sample (see Figure 3). This adjustment avoids the drawback of ‘selecting the best among poor options', making the method more suitable for improvement-oriented contexts (48).

**Step 2:** Calculation of BWM-weighted group utility and maximum regret. For each alternative *A*_*k*_, the BWM-weighted average group utility *S*_*k*_ and maximum regret *R*_*k*_ were computed as:


$$\begin{array}{l} S_{k}=\overset{n}{\underset{j=1}{\sum }}w_{j}r_{kj} \end{array}$$ (7)

$$\begin{array}{l} R_{k}=\max_{j}\left\{r_{kj}|j=1,2,\ldots ,n\right\} \end{array}$$ (8)

These values reflect both overall performance and the weakest dimension of each park.

**Step 3:** Balancing group utility and individual regret. To integrate ‘overall performance' and ‘risk avoidance', a parameter δ (0 ≤ δ ≤ 1) was introduced to define their relative importance of *S*_*k*_ and *R*_*k*_. Following previous studies, δ was set at 0.5 to balance mean utility and maximum regret (64). In the modified VIKOR, the aspired value of both average group utility *S*^*aspired*^ and maximum regret *R*^*aspired*^ are defined as 0, while their worst levels *S*^*worst*^ and *R*^*wost*^ are defined as 1. The synthesized index *V*_*k*_ was then calculated as:


$$\begin{array}{l} V_{k}=\delta \left(S_{k}-S^{\text{aspired }}\right)/\left(S^{\text{worst }}-S^{\text{aspired }}\right)\\ \;+\left(1-\delta \right)\left(R_{k}-R^{\text{aspired }}\right)/\left(R^{\text{wost }}-R^{\text{aspired }}\right)\\ \;=\delta S_{k}+\left(1-\delta \right)R_{k} \end{array}$$ (9)

**Step 4:** Determination of improvement priorities. When decision-makers aim to improve the overall performance of park *A*_*k*_, the standardized gap scores *r*_*kj*_ are multiplied by the BWM-derived weights *w*_*j*_. This produces the weighted gap value for park *A*_*k*_ under criterion *j*, denoted as *g*_*kj*_:


$$\begin{array}{l} g_{kj}=w_{j}\cdot r_{kj} \end{array}$$ (10)

**Step 5:** Ranking of alternatives. Based on the comprehensive index *V*_*k*_ from Step 3, the nine community parks were ranked from best to worst, as shown in Table 4. Combined with the improvement priorities *g*_*kj*_ in Step 4, this evaluation provides quantitative and actionable guidance for enhancing community parks in Xiangzhou District with respect to mitigating depression among older adults.

**Table 4:** Modified VIKOR result.

**No. of cases**	**The specific gaps of each criterion**								**The synthesized Gap**	**Ranking**
	**Access points**	**Pathways**	**Facilities for different activities**	**Open space**	**Amenities**	**Natural features**	**Sunlight**	**Incivilities**		
1	0.050	0.042	0.073	0.067	0.076	0.046	0.036	0.026	0.245	7
2	0.048	0.045	0.080	0.077	0.072	0.040	0.034	0.027	0.252	8
3	0.039	0.037	0.057	0.039	0.054	0.035	0.026	0.024	0.184	2
4	0.039	0.032	0.075	0.035	0.062	0.032	0.031	0.019	0.200	3
5	0.030	0.029	0.046	0.013	0.055	0.040	0.030	0.019	0.158	1
6	0.036	0.028	0.059	0.074	0.059	0.043	0.033	0.019	0.213	6
7	0.042	0.041	0.070	0.038	0.057	0.044	0.030	0.024	0.208	4
8	0.040	0.039	0.063	0.055	0.057	0.050	0.037	0.020	0.212	5
9	0.050	0.042	0.073	0.067	0.076	0.046	0.036	0.026	0.329	9

## 4 Results and discussion

### 4.1 Theoretical implications

The NGST was originally developed to evaluate the overall environmental quality of community parks (63). However, empirical evidence has shown that many of the environmental dimensions it encompasses are also closely associated with the alleviation of depressive symptoms among older adults (8, 12, 33, 60, 67, 68, 70, 74–81, 87–90). Building on this foundation, the present study expanded and refined the NGST framework by incorporating a mental health-oriented perspective. Eight criteria were established, and their relative weights under the goal of ‘improving depression among older adults' were recalibrated using expert knowledge and the BWM, in order to reveal the shifts in criterion priorities across different evaluative perspectives.

The results indicate substantial differences between the new weighting structure and the original NGST. In the NGST, *Incivilities* had the highest weight (24.0%), whereas in the present study, its importance declined considerably (10.6%, ranking sixth among eight), suggesting a relatively limited importance about elderly mental health. Conversely, Recreation Facilities, which had the lowest weight in the NGST (16.26%), emerged as far more important in this study. The related criteria, *Open Space* and *Facilities for Different Activities*, ranked first (16.3%) and second (14.3%), respectively. This shift reflects a transition of focus from ‘environmental quality assessment' toward ‘psychological health intervention'. In addition, *Amenities* and *Natural Features* consistently maintained high importance in both frameworks (NGST: 22% and 20%; this study: 13.5% and 14.0%), demonstrating their sustained significance in park experience and emotional restoration. Meanwhile, Access held a relatively low weight in the NGST (18.0%, second to last), and its corresponding criteria, *Access Points* and *Pathways*, also ranked lowest in this study (10.37% and 10.04%), indicating similar prioritization in both perceptions of park quality and psychological restoration.

According to ART and SRT, *Natural Features* can directly promote psychological relaxation and emotional restoration through visual and sensory channels (19, 24, 91), while conditions such as *Open Space, Activity Facilities*, and *Amenities* indirectly mitigate depression by encouraging physical activity and social interaction (24, 71). In contrast, *access points* and *Incivilities* exert relatively limited effects on mental restoration, which explains their lower weights. Notably, the newly introduced criterion, *Sunlight*, obtained a moderate weight (11.0%, ranked fifth), consistent with recent empirical findings showing that adequate sunlight exposure enhances environmental perception and social interaction among older adults, thereby contributing positively to emotional recovery (71, 81).

Overall, this study systematically revised the traditional NGST framework within the context of improving depression among older adults, developing a more mental health–oriented and context-sensitive weighting system. The proposed framework provides new insights for the evaluation and design of age-friendly community parks.

Beyond criteria weights, this study further examined the characteristics of several alternative ranking methods that are frequently compared with the modified VIKOR method, including Simple Additive Weighting (SAW), the classical VIKOR, Preference Ranking Organization Method for Enrichment Evaluation II (PROMETHEE II), and the Technique for Order Preference by Similarity to the Ideal Solution (TOPSIS) (92, 93)(as shown in Table 5). Experts' direct judgments of overall performance were incorporated, and Spearman correlation analysis was employed to identify both the commonalities and differences among these approaches (as shown in Table 6).

**Table 5:** The comparison of different ranking methods.

**No. of cases**	**Modified VIKOR**		**VIKOR**		**SAW**		**TOPSIS**		**PROMETHEE II**		**EXPERT**	
	**Performance**	**Rank**	**Performance**	**Rank**	**Performance**	**Rank**	**Performance**	**Rank**	**Performance**	**Rank**	**Performance**	**Rank**
1	0.245	7	0.687	8	0.586	3	0.468	3	−1.816	3	6.261	2
2	0.252	8	0.633	7	0.577	2	0.410	2	−1.853	2	6.522	3
3	0.184	2	0.159	2	0.690	8	0.761	8	1.873	8	7.391	6
4	0.200	3	0.378	6	0.676	7	0.701	7	1.270	6	7.565	7
5	0.158	1	0.000	1	0.740	9	0.937	9	3.252	9	8.087	9
6	0.213	6	0.359	4	0.649	5	0.542	4	1.305	7	7.783	8
7	0.208	4	0.370	5	0.655	6	0.680	6	0.576	5	7.261	5
8	0.212	5	0.305	3	0.641	4	0.608	5	0.482	4	6.913	4
9	0.329	9	1.000	9	0.463	1	0.027	1	−5.089	1	5.043	1

**Table 6:** The results of Spearman correlation analysis.

	**Modified VIKOR**	**VIKOR**	**SAW**	**TOPSIS**	**PROMETHEE II**	**EXPERT**
Modified VIKOR	1	0.833^**^	0.983^**^	1.000^**^	0.900^**^	0.800^**^
VIKOR	0.833^**^	1	0.817^**^	0.833^**^	0.867^**^	0.783^*^
SAW	0.983^**^	0.817^**^	1	0.983^**^	0.950^**^	0.867^**^
TOPSIS	1.000^**^	0.833^**^	0.983^**^	1	0.900^**^	0.800^**^
PROMETHEE II	0.900^**^	0.867^**^	0.950^**^	0.900^**^	1	0.933^**^
EXPERT	0.800^**^	0.783^*^	0.867^**^	0.800^**^	0.933^**^	1
Average correlation	0.920	0.856	0.933	0.920	0.925	0.864

^*^ and ^**^ indicating statistical significance at the 5% and 1% levels, respectively.

Spearman correlation results showed that SAW, PROMETHEE II, TOPSIS, and modified VIKOR were more consistent with expert rankings (=0.8), while traditional VIKOR exhibited lower consistency. This indicates that most ranking approaches can replicate expert intuition to some extent. From a decision-making philosophy perspective, different methods embody distinct logics.

From a decision-philosophical perspective, different MCDM methods embody distinct logical mechanisms. The SAW method is based on a simple and intuitive weighted summation, directly aggregating the performance scores of each criterion. In contrast, TOPSIS determines the relative closeness of each alternative by calculating its Euclidean distance to the positive and negative ideal solutions. Previous studies have reported a high degree of similarity between SAW and TOPSIS results (94), and this study reconfirms that finding, with a correlation coefficient as high as 0.983. Traditional VIKOR ranks alternatives solely according to their proximity to the ideal solution and introduces a compromise mechanism between individual criteria and overall performance, which is considered to facilitate convergence toward the ideal solution (93). However, this study found that traditional VIKOR yielded the lowest mean correlation coefficient with other methods (0.856), suggesting that its ranking logic differs substantially from the others. PROMETHEE II determines rankings through pairwise outranking relationships among alternatives, thereby providing a more comprehensive reflection of their relative advantages and disadvantages. Nevertheless, its computational complexity increases under multi-alternative conditions (95). Despite this, the results of this study show that PROMETHEE II achieved the highest agreement with expert judgments, indicating its strong capability in capturing human decision preferences.

Compared with these methods, the Modified VIKOR exhibits a distinctly different decision-making perspective. It extends the ‘realistic feasible ideal level' adopted by TOPSIS and traditional VIKOR into a ‘theoretical aspiration level', making it more suitable for improvement-oriented analyses (48). In addition, the method introduces a mechanism for calculating the priority of criterion-level improvements across alternatives, further enhancing its ability to identify critical weaknesses and formulate targeted improvement strategies (59). Notably, this study found that the ranking results of the Modified VIKOR and TOPSIS were completely identical, with a correlation coefficient of 1.000.

Opricovic and Tzeng (93) pointed out that although VIKOR and TOPSIS differ fundamentally in their aggregation logic, their ranking results diverge significantly only within certain regions of the decision space. Shekhovtsov and Salabun (96) further demonstrated that when the number of alternatives is small, the ranking discrepancies between the two methods tend to diminish. These findings help explain the results of this study: given the limited number of cases and the relatively homogeneous distribution of criterion performances, the distinction between the two methods was not fully manifested, leading to identical ranking outcomes. Collectively, these results deepen the understanding of the methodological positioning and contextual applicability of the modified VIKOR approach.

### 4.2 Practical implications

The results of this study provide specific and systematic design guidelines for improving depressive symptoms among older adults in nine community parks in Xiangzhou District, Zhuhai. The modified VIKOR evaluation revealed that *Facilities for Different Activities* and *Open Space* exhibited the largest performance gaps, representing the most urgent shortcomings to be addressed. This suggests that older adults currently lack sufficient spaces for exercise and social interaction, which hinders the maintenance of positive emotions and psychological wellbeing. To address this issue, it is recommended to prioritize the addition of moderately distributed activity nodes (e.g., fitness equipment, chess tables, and shaded rest areas) within the existing spatial structure, while enhancing spatial openness and accessibility to encourage physical activity and social engagement.

Secondly, the overall performance of the *Amenitie*s criterion was relatively weak (0.055–0.076), indicating insufficient provision of basic facilities such as seating, trash bins, and lighting. This shortage can lead to user inconvenience and environmental disorder. These low-cost yet high-efficiency amenities should therefore be prioritized to improve daily accessibility and comfort. For example, installing continuous seating systems along pathway junctions or landscape nodes, enriching layered nighttime lighting, and maintaining appropriate distances between trash bins and rest areas can significantly enhance user experience.

In addition, several community parks (e.g., Cases 1, 8, and 9) showed considerable variation in *Natural Features* and *Incivilities*, suggesting a need to optimize vegetation hierarchy, increase shade and floral diversity, and reduce negative stimuli such as litter and noise. Although these improvements are not the most urgent, they can exert sustained positive effects on emotional stability and the mitigation of depression among older adults.

By contrast, the score differences for *Access Points, Pathways*, and *Sunlight* conditions were relatively small (approximately 0.03–0.05), indicating that basic accessibility and lighting environments are generally adequate. Subsequent efforts should focus on fine-tuning barrier-free design, paving continuity, and localized light adjustments.

Overall, the practical intervention strategy should follow a progressive logic: first addressing the major deficiencies, then optimizing user experience, and finally refining environmental quality. Priority should be given to improving activity spaces, open areas, and public amenities; subsequently, efforts should focus on enhancing natural elements and environmental order; and finally, detailed adjustments to access points, pathways, and sunlight conditions should be implemented. Such a stepwise approach can maximize the support for elderly mental health and community vitality under limited resource conditions.

## 5 Conclusion

With the accelerating pace of population aging, community parks are playing an increasingly vital role in promoting the mental health of older adults. However, most existing park evaluation systems remain environmentally oriented and lack systematic investigation into their effectiveness in alleviating depressive symptoms among the elderly. To address this gap, this study refined the NGST framework by incorporating a mental health-oriented perspective, thereby establishing an evaluation system focused on improving depression among older adults. Eight criteria were reweighted using expert knowledge and the BWM, and the Modified VIKOR technique was employed to conduct an empirical evaluation of nine community parks in Xiangzhou District, Zhuhai, forming a more actionable and improvement-oriented assessment framework.

The results revealed that *Open space, Facilities for Different Activities*, and *Natural Features* hold the highest priority in mitigating depression, whereas *Access Points, Pathways* and *Incivilities* received relatively lower weights, showing a distinct divergence from the original NGST weighting structure. In addition, the application of the modified VIKOR method demonstrated strong potential for diagnosing key deficiencies and formulating targeted improvement strategies, while also exhibiting high consistency with the TOPSIS approach.

Theoretically, this study extends the applicability of traditional community park evaluation frameworks by integrating mental health goals into the assessment of park quality, enriching the interdisciplinary discourse between geriatric medicine and urban design. Methodologically, by introducing BWM and Modified VIKOR, this study broadens the methodological perspective of environmental evaluation and contributes to a more nuanced understanding of the positioning and comparative strengths of multi-criteria decision-making models.

Practically, the findings provide a systematic priority roadmap for the renewal and optimization of community parks. Priority should be given to improving activity and open spaces as well as public amenities, followed by the enhancement of natural features and environmental order, and finally, the fine-tuning of access, pathways, and sunlight conditions. This stepwise strategy can help maximize mental health benefits and community vitality under limited resource conditions, offering feasible decision support for the creation of age-friendly urban public spaces.

Despite its contributions, this study has several limitations. First, it did not directly collect individual-level data from older adults with depression but instead adopted an exploratory expert-based approach to construct the evaluation framework, without conducting experimental analysis or causal inference. Future research should validate these findings through confirmatory empirical studies. Second, the evaluation framework was built on expert judgment rather than purely objective auditing, which imposes higher requirements on evaluators' professional expertise. Future work could develop hybrid frameworks that integrate objective measurements with expert knowledge to enhance generalizability and operational applicability. Additionally, all experts involved were from mainland China and Macao, whose professional experiences may limit the transferability of the derived weights to other cultural and social contexts. Comparative studies across regions and populations are recommended to improve external validity. Finally, this study evaluated only nine community park cases, which may not provide sufficient robustness for methodological comparisons. Future research should increase the sample size to enable a more systematic analysis of differences and applicability across MCDM approaches.

## Data Availability

The raw data supporting the conclusions of this article will be made available by the authors, without undue reservation.
